# Pulmonary CT perfusion robustly measures cardiac output in the context of multilevel pulmonary occlusion: a porcine study

**DOI:** 10.1186/s41747-024-00431-7

**Published:** 2024-03-22

**Authors:** Diogo Silva, Thomas Muders, Karin Wodack, Christian Putensen, Steffen Leonhardt, Robert Siepmann, Benjamin Hentze, Sebastian Reinartz

**Affiliations:** 1 Medical Information Technology (MedIT), RWTH Aachen University, Aachen, Germany; 2 Department of Anaesthesiology and Intensive Care Medicine, University Bonn, Bonn, Germany; 3 Department of Diagnostic and Interventional Radiology, Uniklinik RWTH Aachen, Aachen, Germany

**Keywords:** Cardiac output, Heart ventricles, Perfusion, Pulmonary artery, Tomography (x-ray computed)

## Abstract

**Background:**

To validate pulmonary computed tomography (CT) perfusion in a porcine model by invasive monitoring of cardiac output (CO) using thermodilution method.

**Methods:**

Animals were studied at a single center, using a Swan-Ganz catheter for invasive CO monitoring as a reference. Fifteen pigs were included. Contrast-enhanced CT perfusion of the descending aorta and right and left pulmonary artery was performed. For variation purposes, a balloon catheter was inserted to block the contralateral pulmonary vascular bed; additionally, two increased CO settings were created by intravenous administration of catecholamines. Finally, stepwise capillary occlusion was performed by intrapulmonary arterial injection of 75-μm microspheres in four stages. A semiautomatic selection of AFs and a recirculation-aware tracer-kinetics model to extract the first-pass of AFs, estimating blood flow with the Stewart-Hamilton method, was implemented. Linear mixed models (LMM) were developed to calibrate blood flow calculations accounting with individual- and cohort-level effects.

**Results:**

Nine of 15 pigs had complete datasets. Strong correlations were observed between calibrated pulmonary (0.73, 95% confidence interval [CI] 0.6–0.82) and aortic blood flow measurements (0.82, 95% CI, 0.73–0.88) and the reference as well as agreements (± 2.24 L/min and ± 1.86 L/min, respectively) comparable to the state of the art, on a relatively wide range of right ventricle-CO measurements.

**Conclusions:**

CT perfusion validly measures CO using LMMs at both individual and cohort levels, as demonstrated by referencing the invasive CO.

**Relevance statement:**

Possible clinical applications of CT perfusion for measuring CO could be in acute pulmonary thromboembolism or to assess right ventricular function to show impairment or mismatch to the left ventricle.

**Key points:**

• CT perfusion measures flow in vessels.

• CT perfusion measures cumulative cardiac output in the aorta and pulmonary vessels.

• CT perfusion validly measures CO using LMMs at both individual and cohort levels, as demonstrated by using the invasive CO as a reference standard.

**Graphical Abstract:**

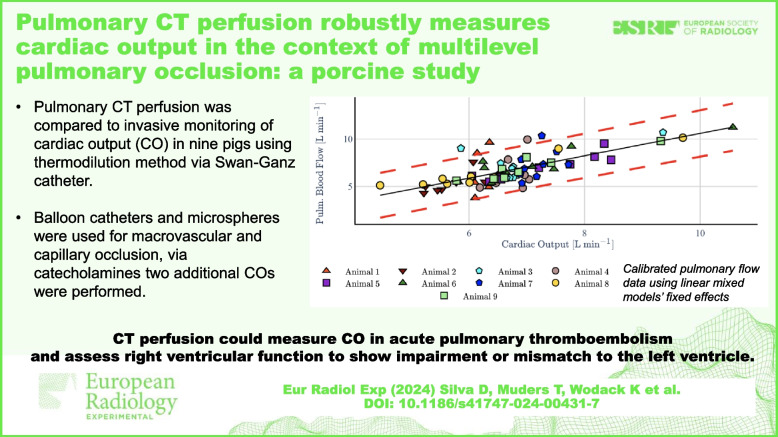

**Supplementary Information:**

The online version contains supplementary material available at 10.1186/s41747-024-00431-7.

## Background

Despite the introduction of computed tomography (CT) into guidelines in 2008 [1], death from pulmonary embolism is on the rise again [2]. Currently, CT-based analysis of contrast-enhancement in the pulmonary artery (PA) is the principal imaging method to exclude PA embolism [3]. In the case of positive finding, the thrombus burden as well as the shape and configuration of the heart chambers are used as image-based biomarkers to assess right ventricular (RV) impairment. Together with laboratory parameters (*e.g.*, N-terminal pro B-type natriuretic peptide, troponin) and echocardiographic findings (*e.g.*, tricuspid valve insufficiency evaluated by tricuspid annular plane systolic excursion, TAPSE), severity classification of circulatory load in the pulmonary vascular bed is assessed. However, the RV function can only be indirectly estimated based on these parameters. During recent years, mortality rates in pulmonary embolism increased in patients 25–65 years (+ 2.1%) [2]. We recall that even with a visually heavy thrombus burden in CT, the clinical presentation can be almost devoid of symptoms, whereas even a visually low thrombus burden may lead to sudden cardiac death or severe hypoxemia [4–6].

Since these indirect approximations insufficiently assess the real impairment of the RV cardiac output (RV-CO) in comparison to the left ventricle (LV)-CO, it has been neglected. However, direct RV-CO measurement could provide a valid risk stratification and a more precise prediction of the mortality rate in the individual patient.

Using CT, RV-CO can in principle be measured by using retrospective ECG-gating technique with complete coverage of the cardiac cycle [7]. This implies a relatively high dose of ionizing radiation accompanied by a time-consuming post-processing procedure to segment the complex RV configuration. Alternatively and analogously to phase-contrast magnetic resonance imaging [8], the iodine flux through a vessel may be quantified by repetitive scans. This corresponds to the RV-CO in the specific vascular territory, *i.e.,* pulmonary trunk. Technically, this is referred to as CT perfusion, which is used for flow or perfusion measurements. It exploits the linear relationship between density upslope and iodine flow rate as a basis for calculating the blood flow of a vessel or in a tissue over time. Clinically, this procedure is successfully implemented for stroke scans [9].

To date, pulmonary CT perfusion showed issues [10] due to outdated CT-scanner technology and high demands on ionizing radiation without proven added value for the patients. Furthermore, only post-acute clinical settings [11] were examined so far.

Against this background, this animal study was conducted in a porcine model, comparing CT perfusion of the aorta and great lung vessels with invasive measurement of RV-CO using a Swan-Ganz catheter. As a correlate for central pulmonary embolism, the corresponding pulmonary vessels were occluded temporarily by a balloon catheter. For mimicking peripheral pulmonary embolism, microspheres were used. The hypothesis was that the RV cardiac output can be quantified by CT perfusion.

## Methods

### Animal preparation

The study was carried out in accordance with the approval of the Competent Authority (LANUV: “Landesamt für Natur, Umwelt und Verbraucherschutz Nordrhein-Westfalen,” Registration Number: 84–02.04.2016.A075) and its Governmental Animal Care and Use Committee. The detailed preparation of the animals is described in Supplementary Method A. Briefly, the animals were intubated with an endotracheal tube and ventilated in volume-controlled mode. The pigs received three large venous sheaths, through which a total of two PACs (Swan-Ganz-Catheter CCO combo 7.5F, Edwards Lifesciences) were inserted into opposite sides of the lung each by the flow-direction method. On the *r*eference side, it was called r-PAC and used for intermittent measurement of RV-CO (Edwards Hemosphere, Edwards Lifesciences); on the *i*ntervention side, it was called the i-PAC and employed to occlude the dependent vascular territories via balloon or by applying microspheres.

### Imaging protocol

A dual-source CT scanner (Somatom Force, Siemens Healthineers, Forchheim, Germany) with a collimation of 96 × 2 × 0.6 mm, a pitch of approximately 1.0, and a tube voltage of 120 kVp was used to examine the entire lung without contrast medium (CM) administration. An electrical impedance tomography belt as part of an electrical impedance tomography device (Pulmovista 500, Draeger, Luebeck, Germany) was used for pulmonary monitoring, which is not addressed in this study. But it obscured the pulmonary trunk and proximal pulmonary arteries for assessment by CT perfusion. To avoid metal artifacts, the examination region was placed caudally to the electrical impedance tomography belt electrodes.

The shuttle mode was used to enlarge the *z*-axis coverage to 113 mm, sacrificing temporal resolution for a wider lung coverage. Tube voltage was set to 70 kVp with a reference tube current of 370 mA. Iodinated CM (iopromide 370 mg I/mL, Bayer Healthcare, Leverkusen, Germany) was administered at 5 mL/s (25 mL), followed by a saline chaser of 50 mL. To assure first pass imaging of the contrast bolus through the pulmonary vasculature, CT perfusion was started simultaneously to the bolus administration.

Non-contrast-enhanced imaging data was discarded prior to evaluation. Reconstructions were calculated with a field of view of 240 × 240 mm, a slice thickness of 1 mm (increment 0.7 mm), and a medium smooth iterative kernel (Br36d) for quantification.

### Experimental setup

The experimental protocol in its entirety was comprised of a total of 10 distinct steps, each of which is delineated by the precise location and configuration of the i-PAC device, either with an inflated or non-inflated balloon, as well as the method of medication or microsphere administration employed. For further details, please refer to Table 1.

**Table 1:** Experimental protocol stages and their objectives

Block type	Target vessels	Unilateral occlusion	Modifiers	Objective
i-PAC inflation	PA	No	-	Baseline
	PA	Yes	-	Effect of central blocking
	PA	Yes	Cardiac output ↑	Effect of increased blood flow
	PA	Yes	Cardiac output ↓	Effect of decreased blood flow
	PA	Yes	Cardiac output ↑↑	Effect of increased blood flow
Microsphere injection	Precapillary	No	-	Baseline
	Precapillary	Yes	+ 1 mL (total 1 mL)	Effect of cumulative peripheral blocking
	Precapillary	Yes	+ 1 mL (total 2 mL)	
	Precapillary	Yes	+ 2 mL (total 4 mL)	
	Precapillary	Yes	+ 2 mL (total 6 mL)	

The table shows an overview of the experimental stages, stating block-type, the targeted vessel, the systemic modifier, and the objective of this experimental part

*PA* Pulmonary artery, *i-PAC* Interventional PA catheter

### Interventions

The process of PA obstruction entailed sequential inflation of the i-PAC device at various positions in accordance with the information provided in Table 1. Verification of the positioning of the catheter or balloon was subsequently confirmed by means of a native CT scan. To provide two additional distinct CO levels, catecholamine infusion (epinephrine 0.02 μg/mL) was administered via an intravenous medication pump (step 7 and step 8). A period of temporary cessation was then implemented to allow for the restoration of normal perfusion (step 9). Following this, stepwise blockade of peripheral pulmonary microcirculation was initiated through the repeated administration of microspheres (Embosphere 75 μm, Merit Medical Systems Inc., South Jordan, Utah, USA). For dosing and sizing purposes, two animals were given microspheres of varying size (ranging from 45 to 450 μm). One milliliter or 2 mL of microspheres were applied via the i-PAC device. In order to assess the combined measurements of CO and CT perfusion under these experimental conditions, the procedures outlined in Table 1 were followed.

### Postprocessing

#### Semiautomatic arterial function (AF) selection

**Fig. 1:**
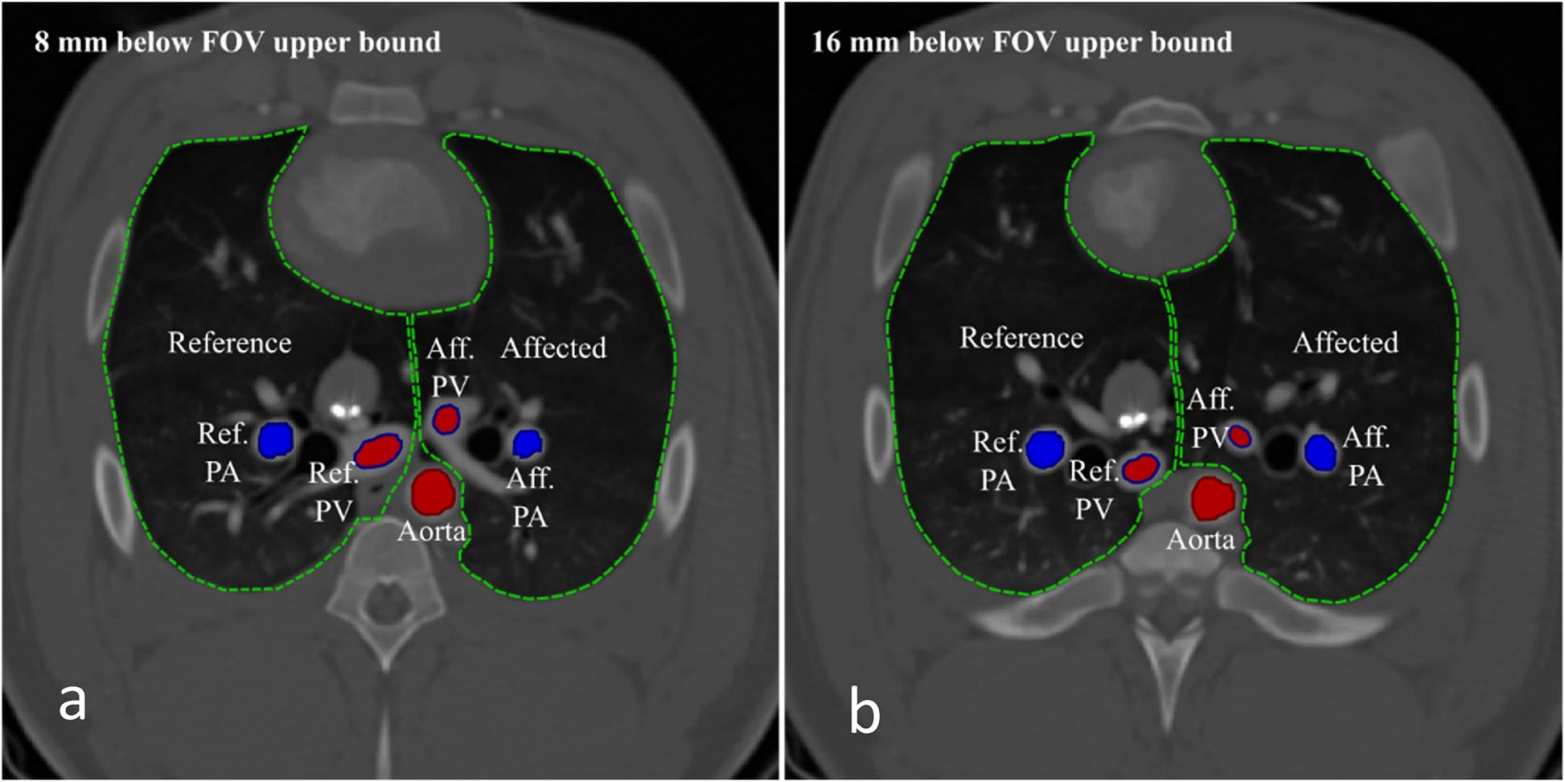
Manual volume of interest (VoI) selection at different slices of the field of view. **a** A slice 8 mm below the upper bound is depicted. **b** A slice 16 mm below the slice in **a**. The lungs are encapsulated by a green intermittent line, while the used VoIs are outlined in red for systemic circulation vessels, in blue for pulmonary vessels. Red filling is used for oxygenated blood, and blue for deoxygenated blood. *Aff.*, Affected; *Ref.*, Reference; *PA*, Pulmonary artery; *PV*, Pulmonary vein

For attaining time-intensity curves from CT perfusion measurements, arterial bolus passage through the PA and the aorta proximal to the r-PAC were evaluated. Therefore, volumes-of-interest (VoIs) were drawn manually inside the aorta and the largest caliber PA vessel cross-section in each side of the lung, incorporating the voxels first traversed by the bolus (Fig. 1). Secondly, a quantile-based selection was applied to retain the voxels enclosing the time-intensity curves with the “most arterial features” as described by Calamante [12]: earliest, steepest, narrowest, largest peak, and area under curve. The algorithm is explained in detail in Supplementary Method C. The AF for each VoI was then calculated by averaging the time-intensity curves of the corresponding surviving voxels. For quality assurance, VoIs in the pulmonary veins were observed.

#### First-pass extraction

**Fig. 2:**
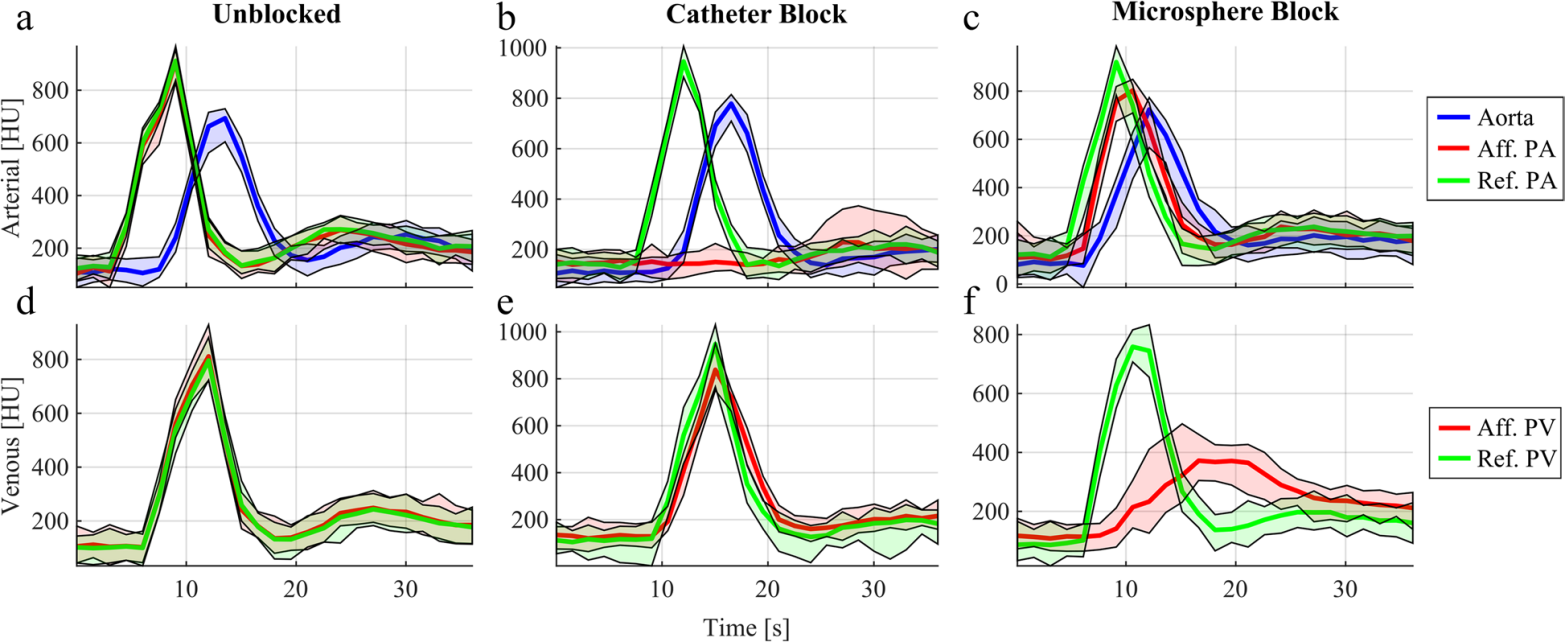
Representative arterial function examples obtained from arterial (**a**, **b**, **c**) and venous (**d**, **e**, **f**) regions of interest described in Fig. 1 without blockage (unblocked, **a** and **d**), under total catheter-induced unilateral blockage of the pulmonary artery (catheter block, **b** and **e**), and microsphere-induced unilateral blockage of pulmonary capillaries (microsphere block, **c** and **f**). Standard deviations are shown with colored shading

To extract the first pass contribution of each AF, a model-based approach accounting for recirculation effects of the CM was employed. Since the AFs were obtained from large caliber vessels, *i.e.,* (pulmonary) arteries, the main sources of recirculation must stem from secondary passages of a not fully dispersed CM bolus. Over many passages, this becomes progressively well mixed in the intravascular volume until a CM concentration steady state. Neither CM leakage to the extravascular space, given the low local permeability of the membrane, nor CM elimination via renal filtration had to be considered, given the relatively short recording. Empirically, these effects chiefly amount to a gamma-variate-shaped first pass ${{\varvec{y}}}_{{\varvec{p}}}$, described by

1 $${y}_{p}\left(t\right)={{y}_{max}\cdot k}_{p}\cdot g\left(t,\alpha ,\beta \right),$$

where ${k}_{p}$ is the fraction of CM molecules localized in the bolus, ${y}_{max}$ is a scalar which amplifies the curve with unitary maximum to the data range, and $\alpha$ and $\beta$ are shape parameters of the gamma-variate function $g$. We note a cumulative intensity portion corresponding to the recirculation ${y}_{r}$

2 $${y}_{r}\left(t\right)={k}_{r}\cdot{\int }_{0}^{t}{y}_{p}\left(\tau \right)\cdot d\tau ,$$

where ${k}_{r}$ is the fraction of CM molecules distributed in the blood. Here, we use the cumulative function of the first pass ${y}_{p}$ to model both the accruing and causal nature of this recirculation effect: a build-up of CM molecules, previously traveling as a local CM concentrate, which progressively detached over many recirculation cycles and mixed with the larger volume of blood. This results in an increase of pixel intensity to a stabilized figure. To ensure conservation of CM mass, ${k}_{p}+{k}_{r}=1$. Finally, the complete model may be obtained by superposing Eqs. (1) and (2) ${y}_{T}\left(t\right)={y}_{p}+{y}_{r}$, (details in Supplementary Method D). A nonlinear least squares algorithm was employed to fit every obtained AF, thereby estimating its parameters and obtaining analytical expressions for the first pass ${y}_{p}$ components, as defined by Eq. (1) and shown in Fig. 2.

#### Pulmonary and aortic blood flow estimation

Using indicator dilution theory [13], blood flow for each AF was calculated from first pass images. A modified Stewart-Hamilton equation

3 $$F=\frac{k}{{\int }_{0}^{\infty }{y}_{p}\left(\tau \right)\cdot d\tau } ,$$

where the flow $F$ is defined as an inverse function of the time-intensity ${y}_{p}$’s integral up to a scaling constant $k$ (L min^-1^ HU min). The latter accounts for inter-subject variability and the conversion factor between Hounsfield units (HU) and CM concentration. This formulation is identical to that used for ground-truth RV-CO measurements, although adapted to the principles of thermodilution and for absolute flow measurement.

If an intensity build-up without wash-out was observed, flow measurements were manually set to zero to avoid abnormally large $F$. Furthermore, blood flow measurements were normalized to facilitate statistical analysis. Pulmonary flow was obtained by adding unilateral flow values from the reference and affected pulmonary VoIs, and aortic flow was calculated from aortic VoIs.

### Statistical analysis

Blood flow data in this study was assumed to be non-independent and structured hierarchically at two levels: animal and cohort. Thus, a linear mixed model (LMM) was used to account for mutually conditioning linear intra-subject dependencies (or random effects) at the animal level and linear inter-subject dependencies (or fixed effects) at the cohort level. Different formulations of LMMs underwent model selection based on the Akaike information criterion and the Bayes factor (Supplementary Method E).

For comparison, the adequacy of simple linear regression was evaluated using Pearson correlation coefficients and 95% confidence intervals (CIs) calculated via the Fisher transform. For the LMM, fixed effects and their standard deviations (SDs) were fitted with a residual maximum likelihood approach, while random effects were estimated using the best linear unbiased prediction approach. Intraclass correlations and supporting CIs were obtained via 1,000 bootstrapping iterations with 80% of the data. The root-mean square error of the LMM and a simple regression model were calculated for further comparison, and a residual analysis was performed using quantile–quantile plots to evaluate the adequacy of the LMM (Supplementary Method F).

Finally, blood flow calculations were calibrated with the reference RV-CO measurements using the fixed effects of the LMM, and a Bland–Altman analysis of the calibrated data was employed to determine the final correlation, estimation bias, and 95% limits of agreement (LoA).

## Results

### Study cohort

**Fig. 3:**
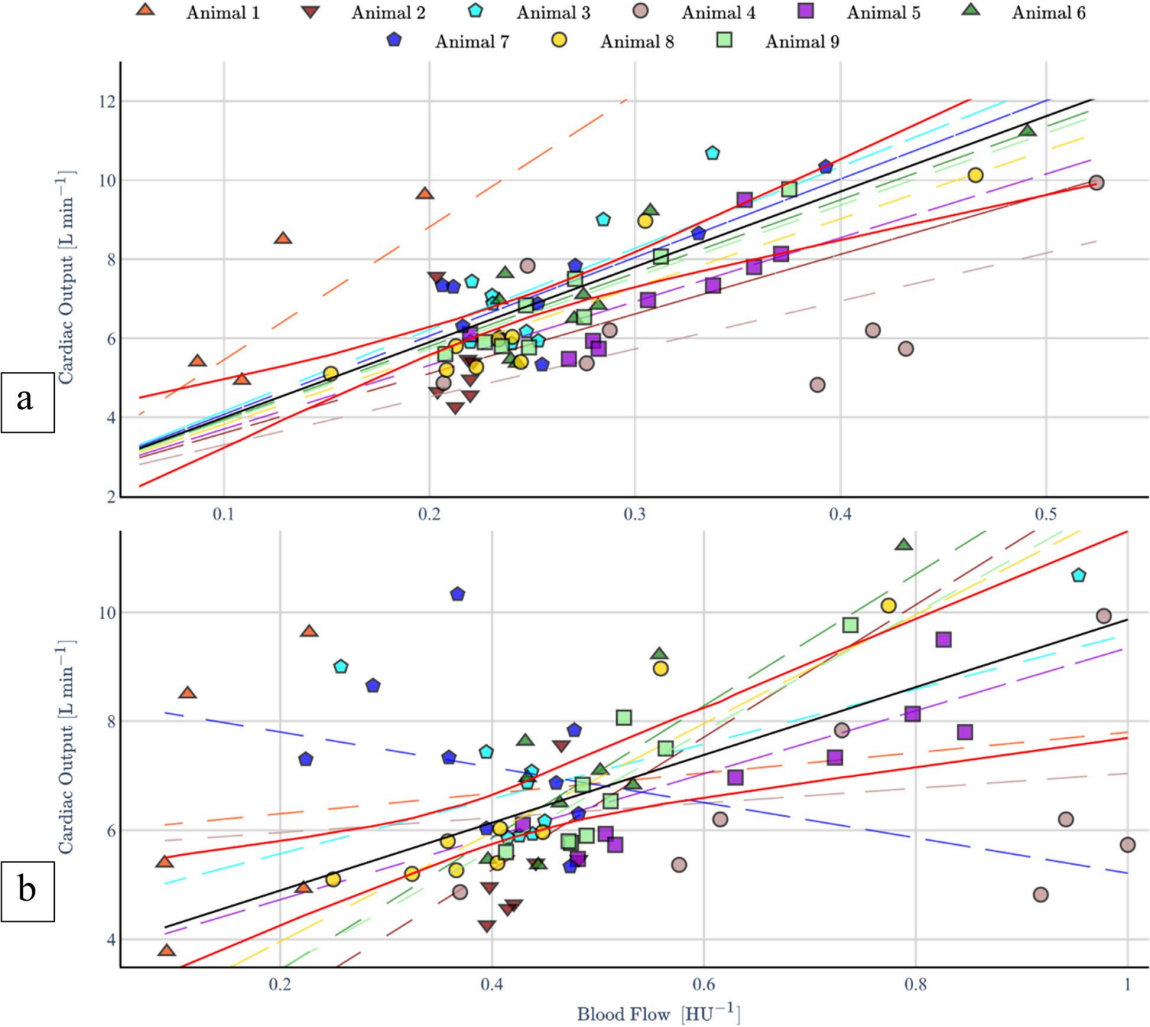
Fitted linear mixed models for aortic flow (**a**) and pulmonary flow (**b**). The fixed effects are displayed as a bold black line, with its 95% confidence intervals in red. Random effects for each individual animal are displayed with differently colored intermittent lines and labelled accordingly. For the aorta, random effects are located tighter to the fixed effects

Fifteen female pigs of Deutsche Landrasse (*Sus scrofa domesticus*) were included in this study, with two used to establish and refine the experimental design. One animal died during the insertion procedure, and measurements on three other pigs were invalid due to catheter misplacement. The remaining nine pigs weighed 62.3 ± 3.8 kg (mean ± SD). A total of 90 CT perfusion flow measurements were evaluated for each aorta, the affected and unaffected PA, and the corresponding AFs are presented in Fig. 3.

### Correlation between vessel flow and CO

Comparing the AFs on the animal- and cohort-level using statistical mathematics, the Pearson correlation coefficients between the measured RV-CO and calculated aortic and pulmonary flow are shown in Table 2. With an average absolute animal-level coefficient of 0.78 ± 0.20 (mean ± SD), and a cohort coefficient of 0.60 (95% CI 0.43–0.73), the aortic flow correlates strongly with RV-CO at the animal level, but not at the cohort level. This is also the case for the pulmonary flow, where a moderate average absolute animal-specific coefficient of 0.68 ± 0.24 and a weak cohort coefficient of 0.37 (95% CI 0.16–0.55) were obtained. In general, correlation figures for the pulmonary flow were weaker than for the aortic flow on the cohort level (*p* ≤ 0.001).

**Table 2:** Comparison between invasive and non-invasive blood flow measurement

Animal	Aortic			Pulmonary		
	Pearson correlation coefficient	95% CIs	*p*-value	Pearson correlation coefficient	95% CIs	*p*-value
1	0.92	[0.12, 0.99]	0.027	0.40	[-0.75, 0.95]	0.509
2	-0.40	[-0.89, 0.50]	0.371	0.70	[-0.11, 0.95]	0.080
3	0.84	[0.41, 0.97]	0.004	0.59	[-0.12, 0.90]	0.094
4	0.49	[-0.33, 0.89]	0.217	0.39	[-0.43, 0.86]	0.334
5	0.82	[0.34, 0.96]	0.007	0.91	[0.60, 0.98]	< 0.001
6	0.87	[0.48, 0.97]	0.003	0.89	[0.55, 0.98]	< 0.001
7	0.80	[0.29, 0.96]	0.010	-0.41	[-0.85, 0.35]	0.270
8	0.92	[0.65, 0.98]	< 0.001	0.94	[0.75, 0.99]	< 0.001
9	0.95	[0.79, 0.99]	< 0.001	0.92	[0.67, 0.98]	< 0.001
Cohort	0.60	[0.43, 0.73]	< 0.001	0.37	[0.16, 0.55]	< 0.001

Correlation between the measured right ventricle cardiac output and the perfusion computed tomography-based calculations of aortic and pulmonary blood flow at the animal and cohort levels

*CI* Confidence interval

### Modeling the relationship between vascular flow and cardiac output using LMMs

**Fig. 4:**
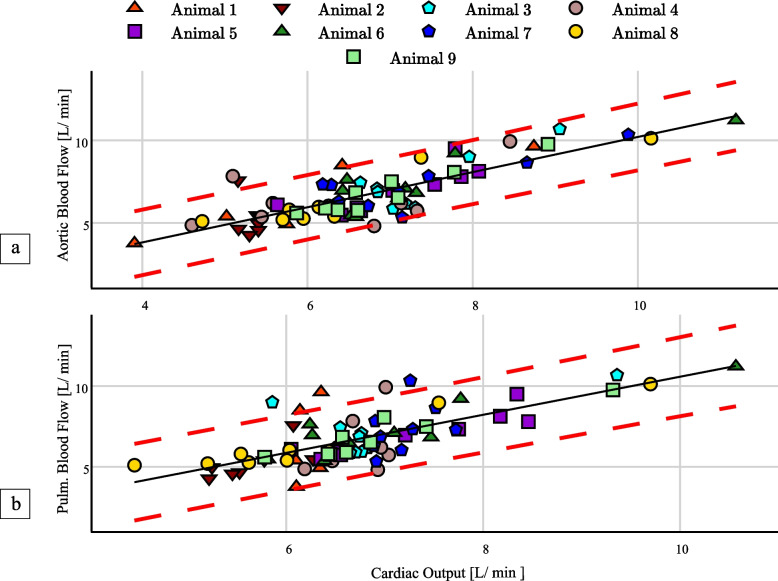
Calibrated aortic flow data (**a**), and pulmonary flow data (**b**), using linear mixed models’ fixed effects

Using the LMMs as relationship model, results for both pulmonary and aortic blood flow as predictors of RV-CO are shown in Fig. 4. The fixed and random effects associated with the model are detailed in Table 3. Both pulmonary and aortic fixed effects capture positive relationship between the random variables, with statistically significant positive slopes of 19.05 ± 6.27 (95% CI 13.00–25.00) and 6.21 ± 6.29 (95% CI 1.41–11.02). Moreover, the pulmonary slope has a larger relative SD than the aortic slope. In general, the LMM parameters were more robustly fitted and estimated for aortic flow, being less variant with narrower CIs. The obtained intraclass correlation coefficients for aortic flow were 0.50 (95% CI 0.47–0.55) and 0.89 (95% CI 0.59–0.98), respectively.

**Table 3:** Estimation of linear mixed model effects

Animal	Aortic			Pulmonary					
	Slope			Intercept			Slope		
	Value	95% CI	SD	Value	95% CIs	SD	Value	95% CI	SD
1	33.63	[22.47, 39.79]	3.14	5.93	[4.41, 7.44]	0.77	1.86	[-1.90, 5.62]	1.92
2	15.08	[11.70, 18.45]	1.72	0.41	[-4.28, 5.09]	2.39	12.16	[2.12, 22.19]	5.12
3	20.62	[18.06, 23.17]	1.30	4.56	[2.47, 6.66]	1.07	5.02	[0.82, 9.23]	2.15
4	12.13	[10.21, 14.04]	0.98	5.68	[3.78, 7.58]	0.97	1.35	[-1.52, 4.22]	1.47
5	16.13	[14.03, 18.22]	1.07	3.57	[1.15, 5.99]	1.24	5.77	[1.64, 9.90]	2.11
6	18.53	[16.33, 20.73]	1.12	1.01	[-2.15, 4.18]	1.61	12.10	[5.86, 18.35]	3.18
7	19.84	[17.43, 22.25]	1.23	8.45	[5.61, 11.28]	1.45	-3.24	[-9.57, 3.09]	3.23
8	17.33	[14.89, 19.75]	1.24	1.96	[-0.39,4.30]	1.20	9.99	[5.05, 14.93]	2.52
9	18.19	[15.79, 20.59]	1.22	1.28	[-2.46, 5.03]	1.91	10.87	[3.55, 18.20]	3.74
Fixed effect		Aortic				Pulmonary			
		Value	95% CI	SD	*p*-value	value	95% CI	SD	*p*-value
Intercept		2.09	[1.04, 3.14]	–	< 0.001	3.65	[1.28, 6.02]	3.11	0.003
Slope		19.05	[13.00, 25.10]	6.27	< 0.001	6.21	[1.41, 11.02]	6.29	0.011

Estimated linear mixed models random and fixed effects describing the relationship between the measured right ventricle cardiac output and the perfusion computed tomography-based calculations of aortic and pulmonary blood flow at the animal and cohort levels, respectively. Since a fixed intercepts model was used for aortic flow, no random effects exist with respect to the fixed intercept

*CI* Confidence interval, *SD* Standard deviation

### Calibration of vessel flow

**Fig. 5:**
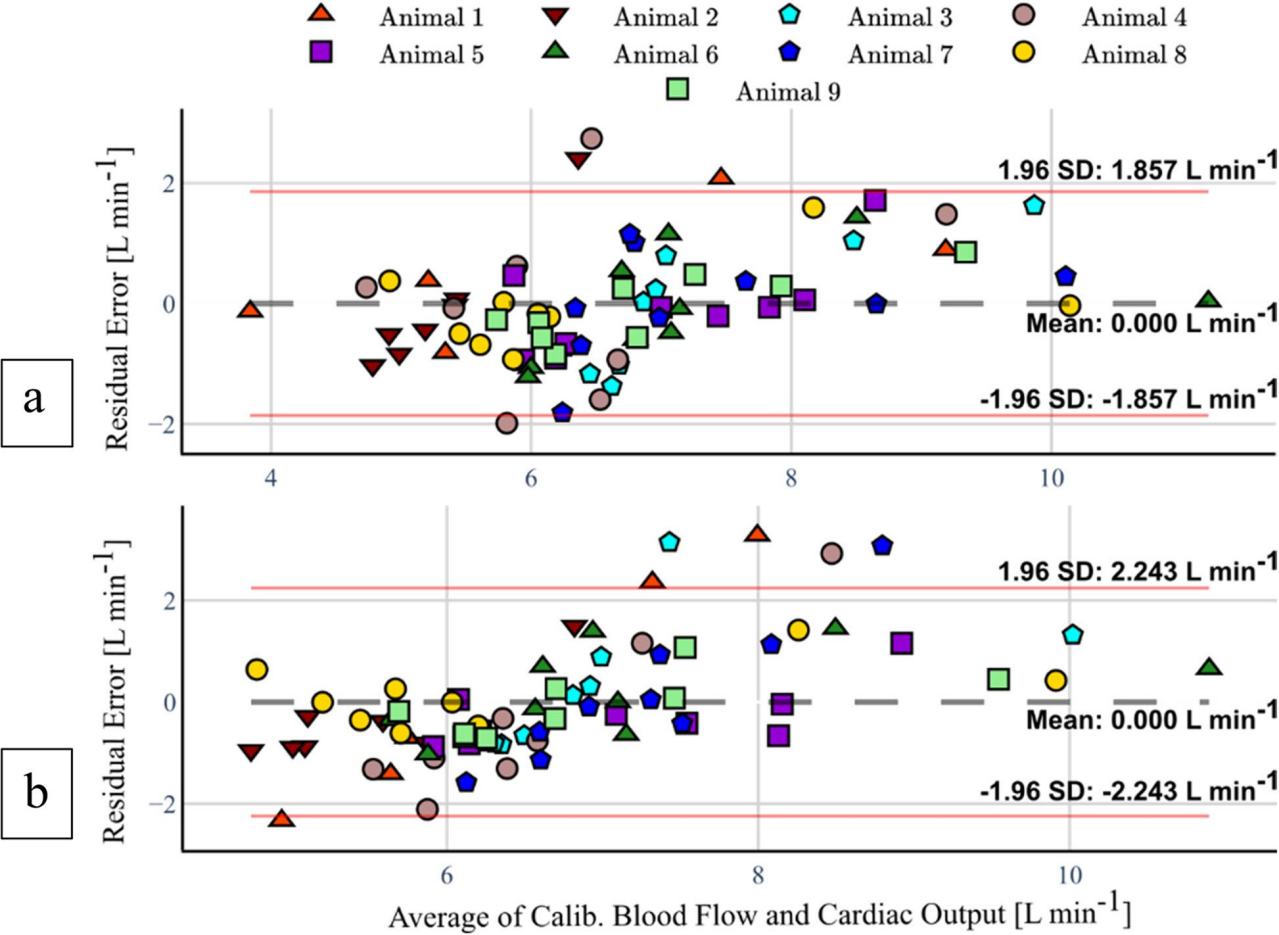
Bland–Altman plot between aortic flow and right ventricle cardiac output measurements (**a**) and pulmonary flow and right ventricle cardiac output measurements (**b**)

The results of LMM-based calibration of the non-invasive blood flow measurements with the fitted fixed-effects is shown in Fig. 5. The intercepts and slopes from a linear analysis of the final calibrated flow measurements and RV-CO are shown in Table 5, while corresponding Pearson correlation coefficients for these are detailed in Table 4. Both aortic and pulmonary results are statistically significant and report strong correlations of 0.82 (95% CI 0.73–0.88) and 0.73 (95% CI 0.6–0.82), respectively. Moreover, root-mean-squared errors of 1.33 and 1.54 were achieved for the aortic and pulmonary data from a simple linear regression calibration, in comparison to reduced figures of 0.95 and 1.15 with the described LMM calibration.

**Table 4:** Comparison between invasive and non-invasive blood flow measurement using linear mixed models

Cohort	Aortic			Pulmonary		
	Pearson correlation coefficient	95% CI	*p*-value	Pearson correlation coefficient	95% CI	*p*-value
	0.82	[0.73, 0.88]	< 0.001	0.73	[0.60, 0.82]	

Cohort level correlation between the measured right ventricle cardiac output and the perfusion computed tomography-based calculations of aortic and pulmonary blood flow after calibration resorting to the fitted linear mixed models

*CI* Confidence interval

### Agreement between CT perfusion and CO

The results of the Bland–Altman analysis between the calibrated non-invasive blood flow calculations and the RV-CO measurements are shown in Fig. 5 and Table 5. A null average residual error was verified for both aortic and pulmonary data, while the 95% LoA lie at ± 1.86 L/min for the former and at ± 2.24 L/min. A slight linear trend in the residuals is also noticeable, although few data points lie beyond the 95% LoA. A detailed residual analysis investigation can be found in Supplementary Method F. Table 4 further specifies that a slight bias is introduced for measurements under induced blockage, and the LoA become wider particularly under catheter blockages.

**Table 5:** Overview of mean differences between invasive and non-invasive blood flow measurements

Group	Aortic			Pulmonary		
	Mean difference	1.96 SD	LoA	Mean difference	1.96 SD	LoA
Cohort	0.00	1.86	[-1.86, 1.86]	0.00	2.24	[-2.24, 2.24]
No block	0.05	1.70	[-1.65, 1.75]	-0.15	1.42	[-1.57, 1.27]
Catheter block	0.39	2.07	[-1.69, 2.46]	0.49	3.01	[-2.52, 3.50]
Microsphere block	-0.39	1.35	[-1.74, 0.96]	-0.38	1.18	[-1.56, 0.80]

Bland–Altman analysis results for the cohort, the no-block and catheter- and microsphere-induced block experimental stages

*LoA* Limits of agreement, *SD* Standard deviation

## Discussion

This porcine study aimed at estimating RV-CO using intravascular CT perfusion flow measurements in the context of pulmonary emboli or obstruction. The suggested LMM calibration approach closely approximated invasive thermodilution RV-CO from the sum of unilateral PA and aortic flow calculations.

While moderate to strong animal-specific linear correlations between pulmonary and aortic flow with RV-CO, 0.68 ± 0.24 and 0.78 ± 0.20, were respectively verified, corresponding cohort-level figures were only weak to moderate, 0.37 (95% CI 0.16–0.55) and 0.60 (95% CI 0.43–0.73). This hints at an inadequacy of simple linear models to connect CT perfusion flow and RV-CO, due to neglected subject-specific effects such as the state of CM dilution at the measurement VoI, the term $k$ from Eq. (4) and blood flow diversion by bifurcations in of the PA trunk or the descending aorta upstream of the measurement plane. Since the latter amounts to subtractive effects, and the former two to multiplicative effects to the measured flow, an LMM model was fitted to the data, allowing for animal-specific intercepts and slopes (random effects) to condition a more robust cohort-level regression (fixed effects). In fact, a statistically significant cohort curve was obtained and used for calibration of CT perfusion RV-CO calculations, yielding strong linear correlation figures of 0.73 (95% CI 0.60–0.82) for pulmonary flow and 0.82 (95% CI 0.73–0.88) for aortic flow. Moreover, non-negligible respective intraclass correlation coefficients of 0.88 (95% CI 0.47–0.98) and 0.48 (95% CI 0.46–0.53) further support the adequacy of the individuality aware LMM approach over simple linear regression. Ultimately, Bland–Altman analysis reported strong agreement between the RV-CO and LMM-calibrated CT perfusion flow, as evidenced by a null bias and relatively narrow 95% LoA of ± 1.86 L/min for the aortic data and ± 2.24 L/min for the pulmonary data. Generally, this study strongly supports the principle that RV-CO can be recovered by adding the unilateral flow measurements. However, since RV- and LV-CO should be similar in hemodynamically stable scenarios, aortic measurements produce a more robust estimate, being influenced by weaker random effects.

Similar studies relating aortic flow from CT perfusion using the Stewart-Hamilton equation and RV-CO obtained through multiple reference methods (*e.g.*, ventricular delineation, aortic peak enhancement) on cohorts of 25 to 50 human subjects [14–17] moderate to strong correlations of 0.60 to 0.82 in a measurement range of 2.82–8 L/min. In particular, Mahnken et al. [14] verified mean difference between both methods of  -2.45 ± 1.30 L min^-1^, as Ludman et al. [16] reported 0.07 ± 2.40 L min^-1^. Using the thermodilution method as reference on 10 dogs, Herfkens et al. [17] and Garrett et al. [18] had reported higher correlations of 0.92 and 0.86, respectively. Pienn et al. [19] additionally calculated flow inside the PA trunk on 18 human subjects and reported for the aorta and PA, respectively, strong correlations of 0.72 and 0.79, and mean differences of 1.00 ± 2.90 L/min, and 0.60 ± 1.50 L/min, in a restricted range of 4–5 L/min.

In contrast to aforementioned works, we used a more sophisticated pre-processing of CT time-intensity curves by implementing AF selection and removing recirculation effects with a modified gamma-variate model, which is shown to mitigate overestimation of blood flow with Stewart-Hamilton’s equation [20]. Moreover, a LMM was employed instead of simple linear regression, allowing for subject-specific imaging and physiological variations to be regarded, which yielded results comparable to the best reported correlations for both aortic and pulmonary flow even without prior calibration of CT number via phantom scans. Finally, our study considered a comparatively wide range of RV-CO measurements (3.78 to 11.23 L/min). Recently, this approach has been demonstrated for pigs in an acute respiratory distress syndrome [21].

Nonetheless, some limitations should be addressed. On the one side, outlying and strongly varying random effects in Fig. 4 and Table 2 for pulmonary flow are likely related to the variable measurement locations in PA branches, only mitigated to a certain extent by the LMM. However, because no related flaws were identified in the procedure, the samples were still considered for the LMM parameterization, producing a pessimistic result of what can be achieved by measuring RV-CO from the PA trunk. On the other side, Bland–Altman analysis showed that there are still some effects in data not explained by a LMM formulation. Optimistically, we estimate that the LoA could improve to ± 1.5 L/min and ± 1.48 L/min, respectively, with a more comprehensive model (Supplementary Method F). Particularly, both aortic and pulmonary measurements seem to be systematically overestimated above 8 L/min, although this might be corrected with data from a more extensive study.

In the future, larger datasets could be gathered to obtain a global mixed-model-based calibration curve which would enable a fast and minimally invasive RV-CO and LV-CO estimation without prior CT number calibration. At some time, RV-CO measurements maybe could participate in the clinical assessment of critically ill patients suffering from acute pulmonary embolism.

### Supplementary Information

**Additional file 1: Supplementary Methods.** A. Animal Preparation. B. Additional Devices. C. Detailed Arterial Function Selection. D. Detailed Fitting Model. E. Linear Mixed Models: Mathematical Formulation and Details on Random Effects. F. Residual Analysis. G. Catheter-induced Pulmonary Vein Backflow. **Supplementary Table S1.** Statistical comparison of considered aortic models. The variable slopes model is here the reduced order model, while the variable slopes and intercepts model is the most complex one. **Supplementary Table S2.** Statistical comparison of considered pulmonary models. The correlated random effects model is here the reduced order model, while the independent random effects model is the most complex one. **Supplementary Figure S1.** Animal level random effects for the aortic (above) and pulmonary (below) models introduced in the main text. **Supplementary Figure S2.** Residual analysis for the aortic (above) and pulmonary (below) models. The residuals are presented as scatter plots in the center, while the corresponding Q-Q plots are shown left, and histograms are shown right. **Supplementary Figure S3.** Bland Altman plot between aortic flow and CO measurements (above), and pulmonary flow and CO measurements (below) reported in the main text after removal of the residual linear trend through orthogonal distance regression. **Supplementary Figure S4.** Series of CT-P images from segmented pulmonary arteries and veins illustrating the backflow phenomenon observed at the PV bifurcation, as a result of blocking blood flow into the left PA. The images are sorted from the earliest (t0) to the latest (t9). Image t3 depicts the drainage of right pulmonary flow into the respective venous vasculature, of which flow becomes apparent in image t4. Between images t4 to t6, the transfer of CM from the right to the left pulmonary veins can be observed, given the absence of a counteracting pressure gradient on the latter region.

## Data Availability

Study data has been stored in facilities of the Institute for Medical Information Technology (MedIT), RWTH Aachen University, Germany. On request, study data is available via medit@hia.rwth-aachen.de.

## Abbreviations

AF: Arterial function
CI: Confidence interval
CM: Contrast medium
CO: Cardiac output
CT: Computed tomography
LMM: Linear mixed model
LoA: Limits of agreement
LV: Left ventricle
PA: Pulmonary artery
PAC: Pulmonary artery catheter
RV: Right ventricle
SD: Standard deviation
