# Supplementary material for: Pulmonary CT perfusion robustly measures cardiac output in the context of multilevel pulmonary occlusion: a porcine study
Source: Eur Radiol Exp. 2024 Mar 22;8:51. doi: 10.1186/s41747-024-00431-7 (PMC10959917; doi:10.1186/s41747-024-00431-7)
Supplement: Supplementary file 1 — Additional file 1: Supplementary Methods. A. Animal Preparation. B. Additional Devices. C. Detailed Arterial Function Selection. D. Detailed Fitting Model. E. Linear Mixed Models: Mathematical Formulation and Details on Random Effects. F. Residual Analysis. G. Catheter-induced Pulmonary Vein Backflow. Supplementary Table S1. Statistical comparison of considered aortic models. The variable slopes model is here the reduced order model, while the variable slopes and intercepts model is the most complex one. Supplementary Table S2. Statistical comparison of considered pulmonary models. The correlated random effects model is here the reduced order model, while the independent random effects model is the most complex one. Supplementary Figure S1. Animal level random effects for the aortic (above) and pulmonary (below) models introduced in the main text. Supplementary Figure S2. Residual analysis for the aortic (above) and pulmonary (below) models. The residuals are presented as scatter plots in the center, while the corresponding Q-Q plots are shown left, and histograms are shown right. Supplementary Figure S3. Bland Altman plot between aortic flow and CO measurements (above), and pulmonary flow and CO measurements (below) reported in the main text after removal of the residual linear trend through orthogonal distance regression. Supplementary Figure S4. Series of CT-P images from segmented pulmonary arteries and veins illustrating the backflow phenomenon observed at the PV bifurcation, as a result of blocking blood flow into the left PA. The images are sorted from the earliest (t0) to the latest (t9). Image t3 depicts the drainage of right pulmonary flow into the respective venous vasculature, of which flow becomes apparent in image t4. Between images t4 to t6, the transfer of CM from the right to the left pulmonary veins can be observed, given the absence of a counteracting pressure gradient on the latter region. [file 41747_2024_431_MOESM1_ESM.docx]

Pulmonary CT perfusion robustly measures cardiac output in the context of multilevel pulmonary occlusion: a porcine study

ELECTRONIC SUPPLEMENTARY MATERIAL

List of Supplementary Materials

**Supplementary Methods:**

A. Animal Preparation

B. Additional Devices

C. Detailed Arterial Function Selection

D. Detailed Fitting Model

E. Linear Mixed Models: Mathematical Formulation and Details on Random Effects

F. Residual AnalysisResidual Analysis

G. Catheter-induced Pulmonary Vein Backflow

**Supplementary Tables:**

Supplementary Table S1. Statistical comparison of considered aortic models. The variable slopes model is here the reduced order model, while the variable slopes and intercepts model is the most complex one.

Supplementary Table S2. Statistical comparison of considered pulmonary models. The correlated random effects model is here the reduced order model, while the independent random effects model is the most complex one.

**Supplementary Figures:**

Supplementary Figure S1. Animal level random effects for the aortic (above) and pulmonary (below) models introduced in the main text.

Supplementary Figure S2. Residual analysis for the aortic (above) and pulmonary (below) models. The residuals are presented as scatter plots in the center, while the corresponding Q-Q plots are shown left, and histograms are shown right.

Supplementary Figure S3. Bland Altman plot between aortic flow and CO measurements (above), and pulmonary flow and CO measurements (below) reported in the main text after removal of the residual linear trend through orthogonal distance regression.

Supplementary Figure S4. Series of CT-P images from segmented pulmonary arteries and veins illustrating the backflow phenomenon observed at the PV bifurcation, as a result of blocking blood flow into the left PA. The images are sorted from the earliest (t_0_) to the latest (t_9_). Image t_3_ depicts the drainage of right pulmonary flow into the respective venous vasculature, of which flow becomes apparent in image t_4_. Between images t_4_ to t_6_, the transfer of CM from the right to the left pulmonary veins can be observed, given the absence of a counteracting pressure gradient on the latter region.

1. Animal Preparation

A veterinary examination of the general condition, weight, lung auscultation and body orifices were carried out and documented on delivery of the animals. For acclimatization, the animals were accommodated for at least one week and given a standard diet (Ssniff pig feed, Ssniff Spezialdiäten GmbH, Soest - 5 % of body weight per day) and water ad libitum. Approval of the experimental protocol was obtained by the local Animal Care and Use Authority (LANUV: “Landesamt für Natur, Umwelt und Verbraucherschutz Nordrhein-Westfalen”, Registration Number: 84-02.04.2016.A075).

Before the experiment, the animals had food restriction for 12 hours, but free access to water. For standardized experimental preparation, atropine 2 mg/kg (Dr Franz Köhler Chemie GmbH, 64625 Bernsheim, Germany) and azaperone 5 mg/kg (Stresnil (R); Lilly Deutschland GmbH, Elanco Animal Health) were administered intramuscularly for premedication purposes. Anesthesia was then induced using propofol 1 mg/kg (Propofol Claris 2%; PHARMORE GmbH) to perform endotracheal intubation. This was maintained by 35 µg/kg/h fentanyl (Rotexmedica Arzneimittelwerk GmbH) and propofol (8 mg/KG x h). Cisatracurium (0.03 mg/kg body weight, repeated every 60 min at 0.1 mg/kg) was administered as needed for muscle relaxation. Then, an 18G indwelling venous cannula and a total of three large venous sheaths (>=10F) were inserted into an ear vein, each sonographically controlled and using the Seldinger technique, mostly bifemoral and/or bicervical. In addition, an arterial sheath was provided femorally to monitor blood pressure. An indwelling urinary bladder catheter facilitated fluid monitoring. Arterial blood pressure was maintained as needed by fluid infusion of NaCl (Baxter, 3542 CE Utrecht, The Netherlands) and glucose 5% (Baxter). A Swan-Ganz Catheter (PAK- Edwards CCO combo 7.5F, Edwards Lifesciences) was introduced into the pulmonal vascular bed to the so-called reference side of the lung via flow direction method. It is equipped with a thermistor for intermittent measurement of CO via the thermodilution method (Edwards Hemosphere, Edwards Lifesciences). A second, non-functional PAK was flow-directed into the contralateral, so-called intervention side, for later occlusion of the experiment-defined vascular territories. Occacionally, a guidewire was used for orthotopic placement.

Ventilation, ECG, arterial and central venous pressure and oxygen were monitored via pulse oximetry throughout the experiment, using a Philips IntelliVue MX700 patient monitor. Anaesthesia was monitored by physicians (TM, KW) experienced in laboratory animal science. After transport to radiology department, the animal was placed supine on the CT gantry on the previously stretched, deflated vacuum mattress. The EIT belt was placed as far as possible cranially on the thorax with the indicator electrodes paramedian to the sternum. The animal was further instrumented for monitoring of CO2, SpO2, invasive arterial blood pressure, central venous pressure, body temperature, ECG and intermittent cardiac output.

Native CT was used to check the position of the EIT electrode belt and the inserted catheters, which were then fixed in place by the vacuum mattress. The body temperature of the animals was kept constant by external warming using a warming blanket.

After completion of the experimental protocol, euthanasia by barbiturates was carried out due to planning as an acute experiment.

1. Additional Devices

An electric impedance tomograph (EIT) monitor (Pulmovista 500, Drägerwerk, AG, Lübeck, Germany) was used synchronously with the other image and data monitors as part of an overall research project. The device features a 16-electrode belt, which is usually placed around the patient’s thorax around the fifth and sixth intercostal spaces and is recognized in CT scans as a high-pixel-intensity artifact akin to the Swan-Ganz catheters.

Quality management:

At the beginning of each trial stage, a short, low-voltage burst of alternating-current at the EIT’s operating frequency was externally injected by an electrode pair placed across the EIT belt, and along the ECG’s lead II using a function generator. This artifact was visible in the continuously obtained data from the EIT device and the patient monitor, allowing their offline synchronization and, thus, leading to a proper temporal reconstruction of the trial’s interventions and all respectively elicited measurements.

1. Detailed Arterial Function Selection

In [1], multiple arterial input function selection approaches are referenced as attempts to filter a VoI based on the most arterial-like features from each voxel comprising it: low TOA, high steepness, low first moment, narrow peak with high maximum, and large AUC. Some approaches also incorporate a metric to filter out noise. For instance, [2] presents an approach relying on sequential quantile-based filtering of AUC and a customized proxy of signal-to-noise ratio, roughness, followed by a clustering technique after which the cluster whose time-intensity curve yielded the lowest first moment was kept.

To select our AFs, we opted for including the entire range of suggested features, and, given the unreliable nature of tailored noise metrics, and the difficulty to reliably estimate the signal-to-noise ratio directly, we preemptively restricted our VoIs to the inner lumina of the vessels away from their respective arterial walls, mitigating the influence of motion artifacts. A single general metric was then derived by assigning equal importance to each feature, normalizing them between 0 and 1 such their sum is unitary

| $\text{score}=I_{\text{peak}}\text{+TOA}+\text{FWHM}_{\text{peak}}+\text{slope}+\text{AUC}+\bar{I},$ | (C1) |
| --- | --- |

where $I_{\text{peak}}$ is the peak intensity value of a voxel’s time-intensity curve, $\text{FWHM}_{\text{peak}}$ is the full-width at half-maximum measure of the curve’s peak, $\text{slope}=I_{\text{peak}}/\text{FWHM}_{\text{peak}}/2$, being defined as the linear slope between the peak’s onset and its highest value $I_{\text{peak}}$, and $\bar{I}$ is the curve’s first moment. To enforce proportionality between the score and “arterialness”, the range of the normalized features $\text{TOA}$, $\text{FWHM}_{\text{peak}}$, and $\bar{I}$ was inverted. Thus, a lower, near-zero $\text{TOA}$ would, for instance, contribute with a higher, near 1 value, to the score. The voxels scoring below the 75^th^ percentile were rejected. Finally, the median absolute deviation method was applied to the AUC of the remaining voxels to remove outliers.

1. Detailed Fitting Model

The gamma-variate function is frequently used to model the CA passage inside of organs as a time-intensity or time-concentration curve

| $g\left( t \vert\alpha,\beta\right)=\beta^{\alpha}\frac{t^{\alpha-1}}{\Gamma(\alpha)}e^{-\beta t}, t>0 \text{and} \alpha, \beta>0,$ | (D1) |
| --- | --- |

where $\alpha$ and $\beta$ are shape-governing parameters, $\Gamma\left( \alpha\right)=(\alpha-1)!$ is the gamma function, $\gamma$ is the incomplete gamma function, and $t\in[0,\infty[$ is time. Its adequacy to these scenarios is sturdily supported by extensive mathematical evidence [3] which highlights its similarity to the transfer function $h_{n}$ of compartment models comprised of a series of $n$ ideally mixing compartments with the same volume $V$ traversed by a constant flow $Q$

| $h_{n}\left( t \right)=\left( \frac{Q}{V} \right)^{n}\frac{t^{n-1}}{(n-1)!}e^{-\frac{Q_{n}}{V}t}, t>0.$ | (D2) |
| --- | --- |

Specifically, under the typical assumption of an ideal instantaneous CA injection, $h_{n}\left( t \right)$ of eq. D2 becomes the time-intensity or time-concentration function of such a tracer kinetics model, drawing obvious physiological parallels with the $g\left( t | \alpha,\beta\right)$ formulation of eq. D1 [4]:

|  | $\alpha=n$ | $\beta=\frac{Qn}{V}.$ | (D3 and D4) |
| --- | --- | --- | --- |

However, although a valid physiological model for the bolus passage through organs with “bucket-like” ($n=1$) behavior, e.g., the lung tissue, or “pipe-like” ($n=\infty$) behavior, e.g. large caliber vessels, it assumes a single first-pass of the CA without additional effects falling under the umbrella term of “recirculation”. Particularly for large caliber vessels, the progressive dilution of the CA particles over the larger intravascular volume as a result of numerous recirculation cycles is especially evident on the VoIs time-intensity curve, and, at latter periods of the recording time (before the onset relevant CA excretion), a large-amplitude event which leads to errors in the hemodynamic parameter estimation if unaccounted for [5]. To describe this process mathematically, we resort to the gamma-variate function $g$ and its cumulative function $G$

| $G\left( t \vert\alpha,\beta\right)=\int_{0}^{t} g\left( \tau\vert\alpha,\beta\right)\cdot d\tau=\frac{1}{\Gamma\left( \alpha\right)}\gamma\left( \alpha,\beta t \right) .$ | D5 |
| --- | --- |

We further assume that a fraction $k_{r}$ of the observed time-intensity curve is due to the accruing concentration due to this recirculation effect, while $k_{p}=1-k_{r}$, is due to the first-pass’ concentration, modelled by the vanilla gamma-variate, arriving at the final model

| $y_{T}\left( t \right)=y_{max}\cdot\left( k_{p}\cdot g\left( \tau\vert\alpha,\beta\right)+k_{r}\cdot\int_{0}^{t} g\left( \tau\vert\alpha,\beta\right)\cdot d\tau\right) ,$ | D6 |
| --- | --- |

with a scaling term $y_{max}$. In this sense, the time-intensity curve of a VoI inside a large caliber vessel can be though of as a superposition of the ideal first-pass with the effect of the consequent and gradual detachment of CA molecules from the travelling bolus volume, leading to a baseline increase cumulating in a pixel intensity steady-state. The $k$ terms enforce the principle of mass implicit in this rationale.

1. Linear Mixed Models: Mathematical Formulation and Details on Random Effects

LMMs are extensions of the traditional linear regression, which accommodate for clusters of dependence in data from a larger population. A typical application for such models is data obtained from repetitive measurements on different subjects, where groups of measurements from the same animal are more similar and, thus, not independent. This is a particular case of hierarchical data with two levels: the population level at the top, and subject level at the bottom. Accordingly, while the LMM assumes a linear trend at the population level, whose governing parameters are called *fixed effects*, it conditions it on individual linear trends at the subject level, whose deviation from the population trend is governed by parameters called *random effects*. Mathematically, the population regression can be expressed as

| $Y=m_{0}\cdot X+b_{0}$ | E1 |
| --- | --- |

where $Y$ is the target variable, $X$ is the predictor variable, $m_{0}$ is the slope and $b_{0}$ the intercept. The slopes $m_{i}$ and intercepts $b_{i}$ of subject $i$’s regression are obtained as normally distributed deviations $u\sim\mathcal{N}\left( 0,\sigma_{m} \right)$ and $v\sim\mathcal{N}(0,\sigma_{b})$ from $m_{0}$ and $b_{0}$, respectively,

| $m_{i}=m_{0}+u_{i},$  $b_{i}=b_{0}+v_{i},$ | E2 |
| --- | --- |

where $\sigma_{m}$ and $\sigma_{b}$ are the corresponding standard deviations. Ultimately, the parameters $m_{0}$, $b_{0}$, $\sigma_{m}$ and $\sigma_{b}$ are fitted to data in mutually affecting fashion.

In this study, a model selection step was firstly performed to choose the LMM formulations presented in the main text. Given the subtractive and multiplicative effects expected to influence blood flow calculations with the pulmonary data, two random slopes and intercepts LMMs – one with the relaxed assumption of correlated random effects, and another with forcefully assumed independent random effects – were tested. For the aortic data, two LMMs – one with simply random slopes, and another with random slopes and intercepts – were tested, to determine which formulation best described the subtractive and multiplicative effects on blood flow measurements. The Akaike information criterion and the Bayes factor were used to choose the models providing the most insight, while being the least complex, as these would yield lower values on the first metric, and higher values on the latter. Additionally, the p-value was used to test the hypothesis that both models produce different results. Tables S1 and S2 summarize the results of this comparison, where an agreement of both metrics in favor of the reduced models can be observed – variable slopes for aortic data, and correlated random effects for pulmonary data, are favored. Since both p-values reject significant differences between the models, the preference for the reduced forms is confirmed.

Table S1. Statistical comparison of considered aortic models. The variable slopes model is here the reduced order model, while the variable slopes and intercepts model is the most complex one.

| Aortic Model | AIC | Bayes | p-value |
| --- | --- | --- | --- |
| Variable Slope | 241.89 | 68.90 | 0.88 |
| Variable Slope + Intercept | 245.75 | 0.02 | - |

*Abbreviations*: AIC – Akaike information criterion; Bayes – Bayes factor.

Table S2. Statistical comparison of considered pulmonary models. The correlated random effects model is here the reduced order model, while the independent random effects model is the most complex one.

| Pulmonary Model | AIC | Bayes | p-value |
| --- | --- | --- | --- |
| Correlated Effects | 249.50 | 8.60 | 1.00 |
| Independent Effects | 251.50 | 0.12 | - |

*Abbreviations*: AIC – Akaike information criterion; Bayes – Bayes factor.

Figure S1 showcases the random effects of the aortic and pulmonary models presented in the main text for the individual animals as linear regressions whose parameters were estimated using the best linear unbiased prediction (BLUP) method. It is observable that the expected positive linear dependence at the animal level holds for the most part. In some animals, e.g., 3, 6 and 8, outlying measurements are present due to large increases in the CO and estimated blood flow induced by the experiments by the administration of stressor medication. This caused some inconsistencies in the fitting, since, in linear models, the most outlying point tends to have more influence on the slope of the curve.


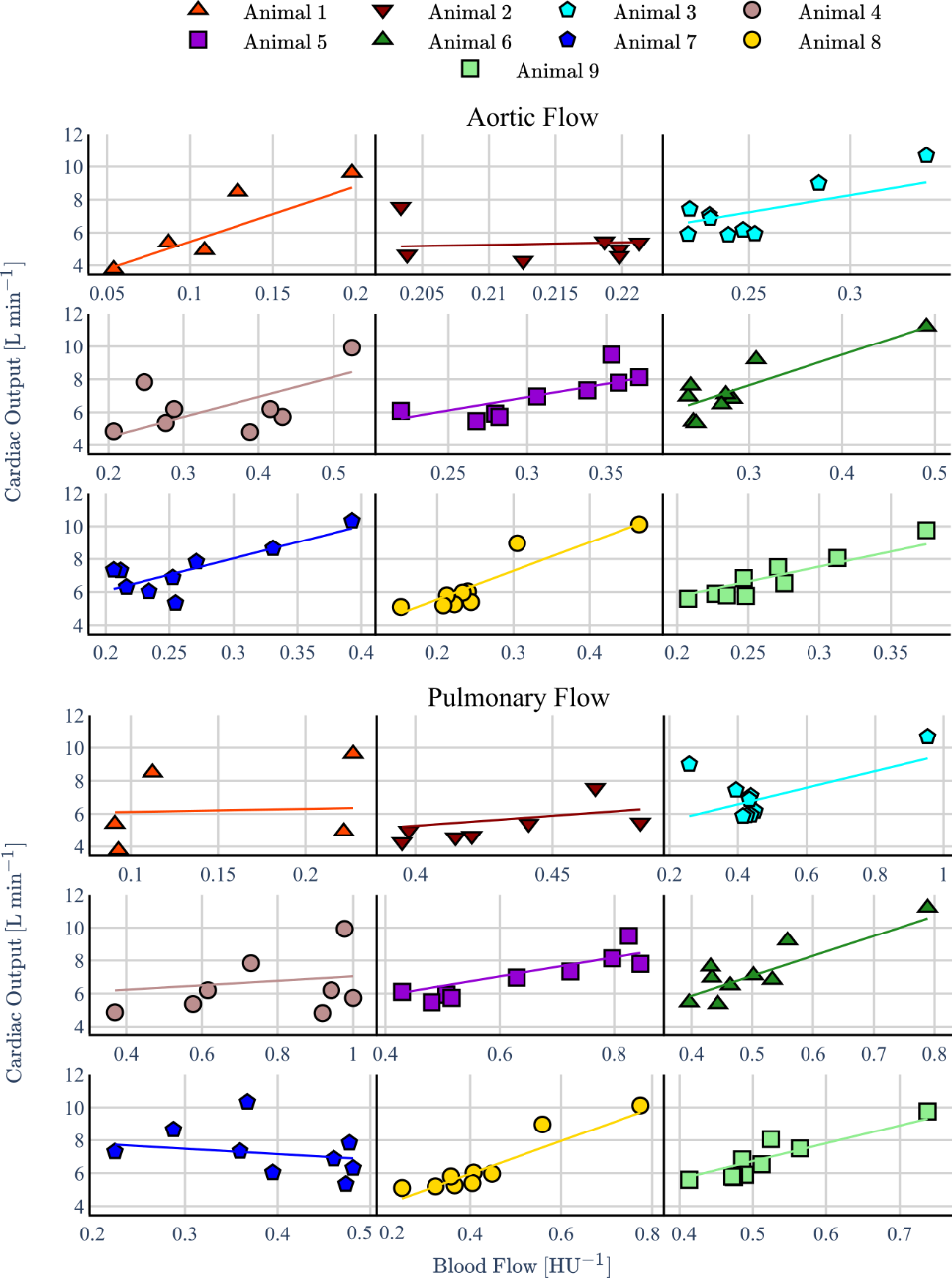


Figure S1. Animal level random effects for the aortic (above) and pulmonary (below) models introduced in the main text.

1. Residual Analysis

A residual analysis of the aortic and pulmonary LMMs presented in the main text can be found in Figure S2. Resorting a visual analysis of the Q-Q plot and the residual histogram, one can observe that the residuals are fairly normally distributed, and, therefore, the assumption of normality is valid. However, a slight tendency for overestimation for high values of CO, and a stronger tendency for underestimation for low values of CO is present. While the former produces a tail effect on the plots, the latter slightly offsets the bulk of the residuals below the zero line.

Since these slight trends in the residuals may amount to an overall positive linear effect unaccounted for by the suggested models, we performed the same the same Bland-Altman analysis presented in Figure 6 of the main text after removing this residual linear trend through orthogonal distance regression. The results are shown in Figure S3, and its reported LoAs represent the level of accuracy which a more comprehensive model to calibrate CT-based blood flow measurements could achieve in a future development. In this case, the improved LoAs would be ± 1.5 L min^-1^ and ± 1.48 L min^-1^ for the aortic and pulmonary data, respectively. The mean value is, in this case, irrelevant, being an unaddressed by-product of the orthogonal distance regression correction.


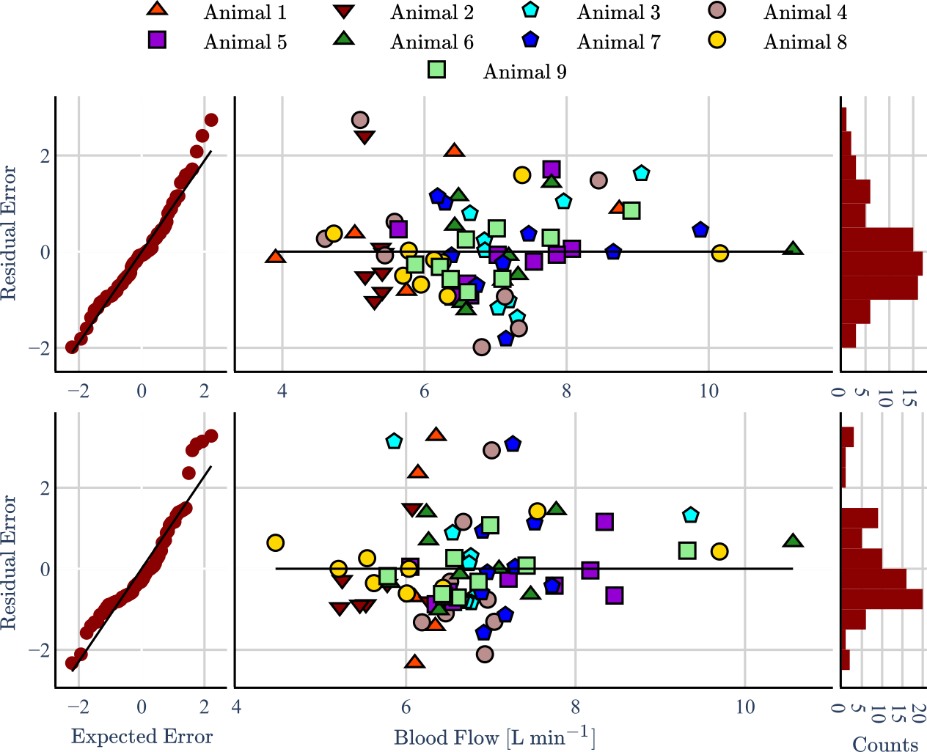


Figure S2. Residual analysis for the aortic (above) and pulmonary (below) models. The residuals are presented as scatter plots in the center, while the corresponding Q-Q plots are shown left, and histograms are shown right.


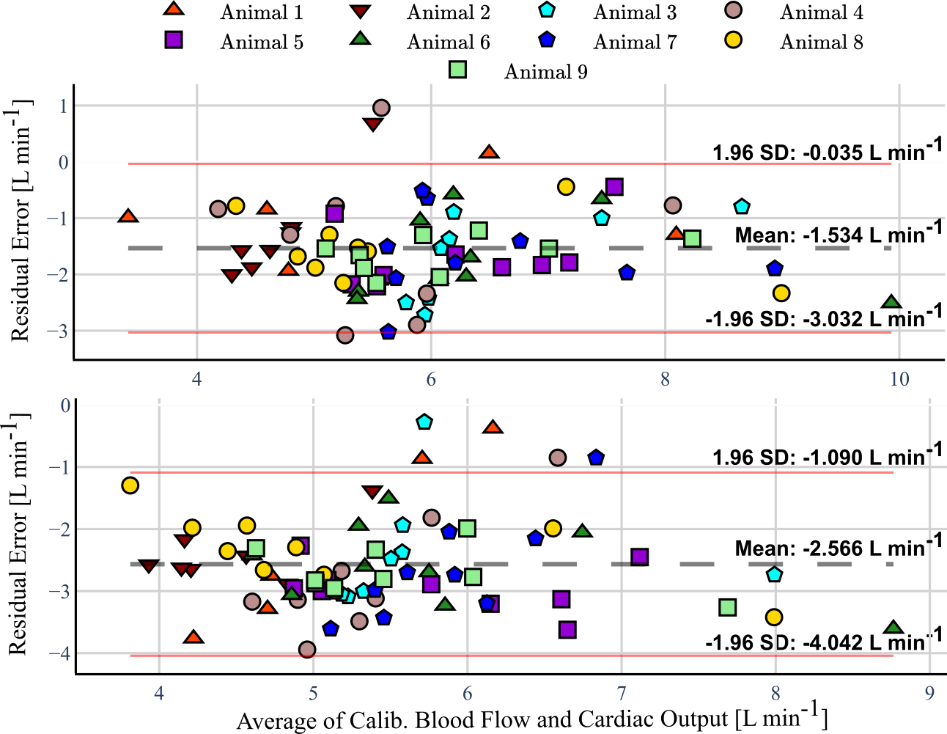


Figure S3. Bland Altman plot between aortic flow and CO measurements (above), and pulmonary flow and CO measurements (below) reported in the main text after removal of the residual linear trend through orthogonal distance regression.

1. Catheter-induced Pulmonary Vein Backflow

Upon the blockage of the PA via the i-PAK, the downstream flow of blood was halted as evidenced by the shrunken and low pixel intensity lumen of the affected PA branch. Figure 3 of the main text further confirms this claim as the AFs of the PA VoI displays an absence of bolus passage. It is, however, observable that the average time-intensity curve of the affected PV VoI does show a bolus passage simultaneous to that of the reference PV VoI. This can be understood by examining the CT-P images over time (Figure S4), where a transfer of the incoming bolus to the PV bifurcation from the reference side is transferred to the affected side in the absence of counteracting pressure caused by the lack of blood flow.


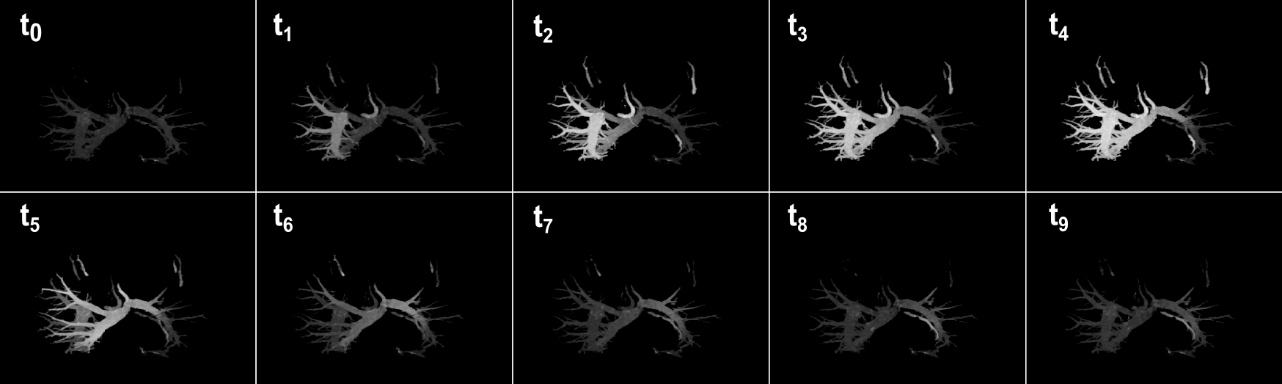


Figure S4. Series of CT-P images from segmented pulmonary arteries and veins illustrating the backflow phenomenon observed at the PV bifurcation, as a result of blocking blood flow into the left PA. The images are sorted from the earliest (t_0_) to the latest (t_9_). Image t_3_ depicts the drainage of right pulmonary flow into the respective venous vasculature, of which flow becomes apparent in image t_4_. Between images t_4_ to t_6_, the transfer of CM from the right to the left pulmonary veins can be observed, given the absence of a counteracting pressure gradient on the latter region.

References

1. Calamante F (2013) Arterial input function in perfusion MRI: A comprehensive review. Prog Nucl Magn Reson Spectrosc 74:1–32

2. Mouridsen K, Christensen S, Gyldensted L, Østergaard L (2006) Automatic selection of arterial input function using cluster analysis. Magn Reson Med 55:524–531

3. Cobelli C, Foster D, Toffolo G (2007) Tracer kinetics in biomedical research: from data to model. Springer Science & Business Media

4. Hentze B, Muders T, Luepschen H, Maripuu E, Hedenstierna G, Putensen C, et al (2018) Regional lung ventilation and perfusion by electrical impedance tomography compared to single-photon emission computed tomography. Physiol Meas 39:065004

5. Patil V, Johnson G (2011) An improved model for describing the contrast bolus in perfusion MRI. Med Phys 38:6380–6383
